# Symptomatic SARS-CoV-2 Reinfection in a Healthy Healthcare Worker in Italy Confirmed by Whole-Genome Sequencing

**DOI:** 10.3390/v13050899

**Published:** 2021-05-12

**Authors:** Daniela Loconsole, Anna Sallustio, Marisa Accogli, Francesca Centrone, Daniele Casulli, Antonino Madaro, Ersilia Tedeschi, Antonio Parisi, Maria Chironna

**Affiliations:** 1 Department of Biomedical Sciences and Human Oncology-Hygiene Section, University of Bari, 70124 Bari, Italy; daniela.loconsole@uniba.it (D.L.); accoisa@gmail.com (M.A.); francesca.centrone.fc@gmail.com (F.C.); 2 Azienda Ospedaliero-Universitaria Consorziale Policlinico di Bari, 70124 Bari, Italy; annasallustio@libero.it (A.S.); daniele.casulli@hotmail.com (D.C.); 3 Department of Prevention, Local Health Unit of Bari, 70124 Bari, Italy; antonino.madaro@asl.bari.it; 4 Covid-Unit, Hospital F. Miulli, Acquaviva delle Fonti, 70124 Bari, Italy; tedersy@gmail.com; 5 Istituto Zooprofilattico Sperimentale della Puglia e della Basilicata, 71121 Foggia, Italy; antonio.parisi@izspb.it

**Keywords:** reinfection, COVID-19, SARS-CoV-2 infection, whole-genome sequencing, clades-lineages, anti-SARS-CoV-2 IgG antibodies

## Abstract

This study describes a case of SARS-CoV-2 reinfection confirmed by whole-genome sequencing in a healthy physician who had been working in a COVID-19 hospital in Italy since the beginning of the pandemic. Nasopharyngeal swabs were obtained from the patient at each presentation as part of routine surveillance. Nucleic acid amplification testing was performed on the two samples to confirm SARS-CoV-2 infection, and serological tests were used to detect SARS-CoV-2 IgG antibodies. Comparative genome analysis with whole-genome sequencing was performed on nasopharyngeal swabs collected during the two episodes of COVID-19. The first COVID-19 episode was in March 2020, and the second was in January 2021. Both SARS-CoV-2 infections presented with mild symptoms, and seroconversion for SARS-CoV-2 IgG was documented. Genomic analysis showed that the viral genome from the first infection belonged to the lineage B.1.1.74, while that from the second infection to the lineage B.1.177. Epidemiological, clinical, serological, and genomic analyses confirmed that the second episode of SARS-CoV-2 infection in the healthcare worker met the qualifications for “best evidence” for reinfection. Further studies are urgently needed to assess the frequency of such a worrisome occurrence, particularly in the light of the recent diffusion of SARS-CoV-2 variants of concern.

## 1. Introduction

The ongoing COVID-19 pandemic has affected almost one hundred and forty million people worldwide, with nearly three million deaths [1]. Over the past few months, several cases of clinical recurrence of COVID-19 have been reported [2,3,4]. The possible causes of SARS-CoV-2 relapse are still poorly understood. It has been hypothesized that cases of recurrence might occur when suboptimal control of the infection allows for a second episode of viral replication or reactivation of the virus within sanctuaries [5]. COVID-19 recurrences should be distinguished from secondary complications, persistent infections, or reinfections [5,6]. Reinfections are defined as the detection of SARS-CoV-2 RNA in respiratory samples of patients with or without respiratory symptoms more than 90 days after the initial infection [7]. In most cases, the second infection is less severe than the first episode [8,9], but in some cases, patients show more severe symptoms upon reinfection than at the first occurrence [10,11].

Questions about whether the immune response after the first infection could protect against reinfection are still unresolved, as immunity after SARS-CoV-2 infection is not yet well understood [12]. For common cold coronaviruses, loss of immunity frequently occurs within 12 months after primary infection [13]. Patients with a previously confirmed SARS-CoV-2 infection are likely to be protected against reinfection for several months, as the infection results in the generation of neutralizing antibodies [14]. In most cases, this occurs within 2 weeks of infection [15], with a more rapid and stronger neutralizing response in severe cases than in mild cases [16]. However, some studies have shown that antibody titers start to decline as early as 1–2 months after the acute infection [17]. Due to this waning immunity, patients with mild COVID-19 disease are considered more susceptible to potential reinfections [17]. Several SARS-CoV-2 reinfections have been documented recently, including in healthcare workers (HCW) and inpatients with no history of clinically significant medical conditions [11,18,19,20,21]. The results of a survey conducted in the EU/EAA countries by the European Centre for Disease Prevention and Control (ECDC) at the end of January 2021, showed that 1.887 suspected cases of reinfection were reported in 2020 and 691 cases were under investigation in 2021 [22]. Despite these reports, cases of reinfection remain rare, although they are likely underestimated [10].

Here, we describe a case of reinfection with the SARS-CoV-2 virus in an HCW in Italy. In addition to reporting key epidemiological, clinical, and serological data, we confirmed the reinfection using whole-genome analysis, which demonstrated genetically distinct SARS-CoV-2 agents.

## 2. Materials and Methods

### 2.1. Epidemiological Surveillance

The COVID-19 national surveillance system in Italy includes integrated microbiological and epidemiological surveillance and is coordinated by the Department of Infectious Diseases at the Istituto Superiore di Sanità (Rome, Italy). All cases of suspected SARS-CoV-2 infection are investigated at the local level. In the Apulia region, a network of laboratories processes respiratory samples from all suspected SARS-CoV-2 infection. All epidemiological information and molecular data from confirmed SARS-CoV-2 cases are uploaded to an online surveillance platform (https://covid-19.iss.it/login.aspx?ReturnUrl=%2f, accessed on 14 February 2021).

### 2.2. Case Definition for SARS-CoV-2 Reinfection

The reinfection was classified according to the CDC criteria as detection of SARS-CoV-2 RNA ≥90 days since the first SARS-CoV-2 infection in a person with or without COVID-19-like symptoms and the availability of paired respiratory specimens from each infection episode [7]. The evidence level for reinfection using genomic data was considered “best evidence” if the strains of the two episodes belonged to different clades, as defined in the Nextstrain and GISAID databases, ideally with other evidence of infection (e.g., high viral titers in each sample, detection of sgmRNA, or viral culture). “Moderate evidence” was considered the presence of >2 nucleotide differences per month in consensus between sequences that met the quality metrics above, ideally coupled with other evidence of actual infection (e.g., high viral titers in each sample, detection of sgmRNA, or viral culture) [7].

### 2.3. RT-PCR and Antibody Testing 

Nasopharyngeal swabs (FLOQSwabs, Copan Italia, Brescia, Italy) were collected from the patient during both episodes as part of routine surveillance and were stored at −80 °C. Samples were analyzed at the Laboratory of Molecular Epidemiology and Public Health of the Hygiene Unit (A.O.U.C. Policlinico, Bari, Italy), which is the coordinator of the Regional Laboratory Network for SARS-CoV-2 diagnosis in the Apulia region. The sample collected during the first episode was retested after the second episode. The RNA was extracted using the MagMAX Viral/Pathogen Nucleic Acid Isolation kit (Thermo Fisher Scientific, Waltham, MA, USA). The molecular test was performed using a three-target (N, ORF1ab, and S genes) commercial multiplex real-time PCR assay from Thermo Fisher Scientific (TaqPath RT-PCR COVID-19 Assay). After the first episode, a chemiluminescent immunoassay that detects IgG against the nucleocapsid protein of SARS-CoV-2 was performed on the patient’s serum samples (Abbott SARS-CoV-2 IgG assay on the Abbott Architect i4000SR; Abbott Diagnostics, Chicago, IL, USA). A signal/cut-off (S/CO) ratio of ≥1.4 was interpreted as reactive, and an S/CO ratio of <1.4 as nonreactive, as per the manufacturer’s instructions. After the second episode, a chemiluminescent microparticle immunoassay (CMIA) that detects IgG against the spike protein of SARS-CoV-2 was performed (SARS-CoV-2 IgG II Quant assay on the Abbott Architect iSystem; Abbott Diagnostics, Chicago, IL, USA). A value of IgG < 50 UA/mL was considered nonreactive and a value of IgG ≥ 50 UA/mL as reactive, according to the manufacturer’s instructions.

### 2.4. Viral Whole-Genome Sequencing 

Whole-genome sequencing was performed using the Ion Torrent platform (Thermo Fisher Scientific, Waltham, MA, USA). Reverse transcription was performed using the SuperScript Vilo cDNA synthesis kit (Thermo Fisher, Waltham, MA, USA). The cDNA was then used for SARS-CoV-2 tiling PCR. The libraries were prepared using the Ion Ampliseq Library kit Plus and the Ion AmpliSeq SARS-CoV-2 RNA custom primers panel according to the manufacturer’s instructions (Thermo Fisher Scientific, Waltham, MA, USA). 

Quality control of Ampliseq reads, as well as their alignment to the complete genome of the SARS-CoV-2 isolate Wuhan-Hu-1, was performed on the Torrent Server of an Ion Torrent S5 sequencer using the default settings. The aligned reads were used for both reference-guided assembly and variant calling. Assembly was performed using the Iterative Refinement Meta-Assembler (IRMA) v.1.3.0.2, which produced a consensus sequence for the samples using a >50% cut-off for calling single nucleotide polymorphisms.

### 2.5. Phylogenetic Analysis 

The phylogenetic analysis was performed using the Nextclade sequence analysis webapp (https://clades.nextstrain.org/, accessed on 14 February 2021). To identify strains that were most closely related to those of the two patient samples, strains in the GISAID database were analyzed (www.gisaid.org, accessed on 14 February 2021). The clade information was described using the GISAID and Nextstrain nomenclature, and the lineage information was described using the Pangolin nomenclature.

## 3. Results

### 3.1. Patient

The patient was a healthy 41-year-old female physician without comorbidities or immunological disorders who had been working in a COVID-19 hospital in the province of Bari, Italy, since the beginning of the pandemic. On 20 March 2020, she presented with strong arthralgia, low-grade fever (T max, 37.5 °C), headache, and diarrhea. The diagnosis was confirmed by a positive SARS-CoV-2 molecular test performed on the nasopharyngeal swab collected on 21 March 2020. The infection was likely acquired as a result of a nosocomial exposure, as an outbreak among HCWs in the ward in which the patient was working occurred at that time. Three days after the onset of symptoms, the patient showed anosmia and ageusia. During the symptomatic phase, paracetamol 1000 mg was administered every 12 h. Symptoms resolved in 3 days, except for the anosmia and ageusia, which lasted until the summer months, along with sleep disorders and impaired concentration. The respiratory samples collected in the weeks following the diagnosis showed positivity for SARS-CoV-2 until 29 April 2020. The first antibody test performed in June 2020 showed an S/CO ratio of 4.49.

As a healthcare worker, between April and December, 2020, the patient was screened every month for SARS-CoV-2 infection and tested negative. On 4 January 2021, a nasopharyngeal swab and a serum sample were collected for routine screening. The molecular test was negative and the antibody test result was 66.5 UA/mL. On 6 January 2021, the patient was scheduled to receive the first dose of the COVID-19 Comirnaty vaccine (Pfizer-BioNTech); however, due to a persistent headache, it was postponed to 11 January 2021. On 7 January 2021, another nasopharyngeal swab was collected and was negative for SARS-CoV-2. The day after, the patient showed symptoms consistent with COVID-19, including a sore throat and headache, but she was afebrile. On 11 January 2021, the patient underwent another nasopharyngeal swab in the morning, and the first dose of the COVID-19 Comirnaty vaccine (Pfizer-BioNTech) was administered in the afternoon. The molecular test was positive for SARS-CoV-2 infection. The epidemiological investigation revealed that the patient acquired the infection as a result of occupational exposure, as she lives alone and had no contact with relatives without personal protective equipment (PPE) or any history of travel. Her immediate contacts were tested, and none became infected. From 14–31 January, the patient reported abdominal pain and diarrhea. The nasopharyngeal swab collected on 23 January 2021 was negative for SARS-CoV-2 infection. Because of the persistence of diarrhea, a rectal swab was collected on 31 January 2021 and was negative for SARS-CoV-2. On 2 February 2021, a second antibody test was performed, and the result was 5545 UA/mL. The second dose of the COVID-19 Comirnaty vaccine (Pfizer-BioNTech) was administered on 4 February 2021.

### 3.2. Genome Analysis 

Molecular tests on the nasopharyngeal swabs collected during the first episode in March 2020 and the second episode in January 2021 were positive for SARS-CoV-2, with a high viral load. Specifically, the first sample collected in March 2020 and retested in January 2021 showed the following Ct values for each target: N gene, 30; ORF1ab, 27; and S gene, 29. The second sample, collected and tested in January 2021, showed a Ct value of 15 for the N gene, 12 for ORF1ab, and 13 for the S gene.

**Figure 1:**
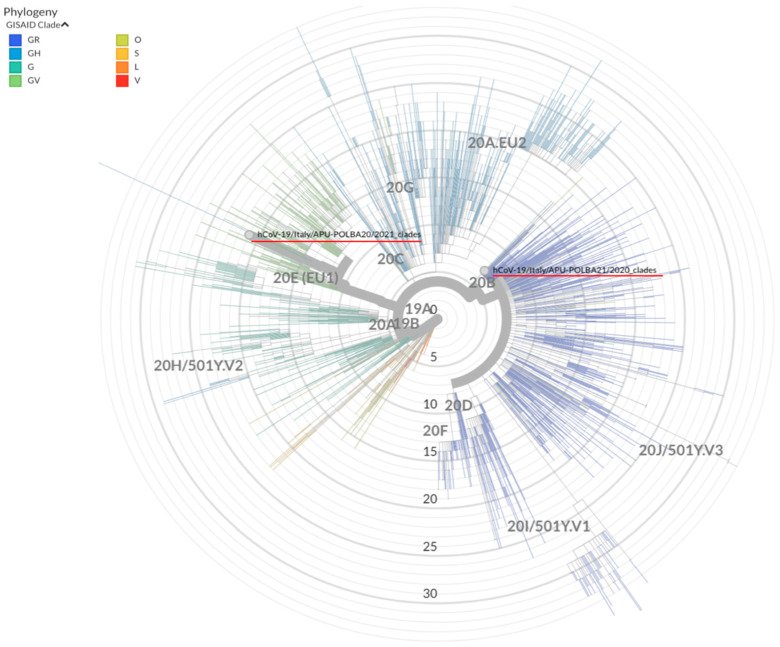
Phylogenetic analysis of SARS-CoV-2/human/ITA/APU-POLBA21/2020 and SARS-CoV-2/human/ITA/APU-POLBA20/2021. Other genomes were retrieved from GISAID (https://www.gisaid.org, accessed on 14 February 2021). Clade information using the GISAID and Nextstrain nomenclatures is shown. The strains of the present study are underlined in red.

Whole-genome sequencing was performed on samples collected during both episodes. The sequences encompassed the entire genome. The strain from the first COVID-19 episode was identified as SARS-CoV-2/human/ITA/APU-POLBA21/2020 (GenBank Accession Number: MW652721), and the strain from the second infection was identified as SARS-CoV-2/human/ITA/APU-POLBA20/2021 (GenBank Accession Number: MW652728). Genomic analysis showed that the strain identified during the first infection belonged to a different clade than the strain identified from the second infection. In particular, the SARS-CoV-2/human/ITA/APU-POLBA21/2020 strain belongs to the GISAID clade GR, Nextstrain clade 20 B, PANGO lineage B.1.1.74, while the SARS-CoV-2/human/ITA/APU-POLBA20/2021 strain belongs to the GISAID clade GV, Nextstrain clade 20 E(EU1), PANGO lineage B.1.177 (Figure 1). The evidence for reinfection therefore met the qualifications for “best evidence.” The mutations identified in the two strains are listed in Table 1. We performed a basic local alignment search tool (BLAST) search of the two genomes. The first SARS-CoV-2 genome was most closely related to strains identified in Spain in March and April 2020, while the second genome was most closely related to strains identified in Spain and France in November and December 2020.

## 4. Discussion

We report a case of SARS-CoV-2 reinfection that occurred in a healthy healthcare worker in Italy. It is still unclear how often reinfections can occur. There are still limited population-level data available that captures the burden of reinfections at the national level, although instances of reinfection with different strains seem to be on the rise [22]. To the best of our knowledge, there is no other reinfection documented with whole-genome sequencing in Italy. In the case described here, several lines of evidence support a case of reinfection instead of recurrence. First, the whole-genome sequence analysis demonstrated that the two strains belong to different clades, and this evidence rules out the hypothesis of persistent viral shedding from the first infection. Moreover, the RT-PCR data showed a high viral load in each sample, which is strong evidence for reinfection according to CDC criteria. Second, the virus identified in the second infection did not harbor any known spike mutation that could have enabled escape from immunity induced by the primary infection. Moreover, approximately 9 months elapsed between the two COVID-19 episodes, which is much longer than the 185 days between infections reported by Selhorst et al. for a Belgian HCW [21].

The patient described here showed mild COVID-19 symptoms in both episodes. This is in accordance with cases reported from Hong Kong [18], Belgium [21], and the Netherlands [19] but in contrast with cases from Ecuador [20] and the UK [11], where patients presented with increased symptom severity in the second infection.

Our patient reported occupational exposure to SARS-CoV-2 in both episodes. The first infection was acquired in the early stage of the pandemic, when the dynamics of the virus transmission were not completely understood and PPE was not available for all HCWs and patients. In the second episode, the exposure was assumed to be occupational because the patient had no close contact with other individuals with COVID-19 and no history of travel. Unfortunately, no SARS-CoV-2 strains from patients hospitalized in January 2021 in the same hospital as the HCW were available for molecular characterization and comparison. Several cases of SARS-CoV-2 reinfection in HCWs have been described, some of which were asymptomatic in both episodes and identified during routine surveillance screenings [23]. As previously described, in the case of healthy HCWs showing mild symptoms in both episodes, reinfection can be surmised due to the prolonged exposure [5,16]. HCWs are at much higher risk of repeated exposure to the virus compared with the general population, and the occurrence of these cases of reinfection despite the use of PPE confirms that HCWs are a priority for COVID-19 vaccination [24].

Immunity to SARS-CoV-2 involves both humoral and cell-mediated responses, but its ability to protect from reinfection is still uncertain [25], and it is not possible to determine whether a protective immune response developed using conventional tests for SARS-CoV-2-specific antibodies [20]. Vaccine-induced immune responses are more consistent than those that are triggered naturally [26], so it can be speculated that vaccinating a large proportion of the population could also prevent reinfections.

The emergence of variants of SARS-CoV-2 with increased virulence is a new matter of concern, particularly in relation to possible reinfection. The three emerging variants of concern (VOCs) that were recently identified are associated with a rise in the incidence and mortality of COVID-19 [27]. In Europe, community spread of the three VOCs has led to higher hospitalization and death rates across all age groups [27]. This spread is worrisome also in the light of the reported reduced neutralizing capacity of SARS-CoV-2 antibodies against VOCs, in particular those harboring the E484K mutation [27]. Moreover, the increased transmissibility of these strains poses concern about these variants as a possible cause of reinfections as well as postvaccination infections [10]. In the Apulia region, the VOC 202012/01 was first identified at the end of December 2020 [28], and its prevalence in Italy in March 2021 has been estimated to be 86.7% [29]. Further investigation is urgently needed to monitor vaccine effectiveness against these VOC [10].

Our study has some limitations. First, it was not possible to fully assess the effectiveness of the immune response during the two episodes of SARS-CoV-2 infection, as neutralizing antibody titers were not measured. Moreover, viral culture of the two SARS-CoV-2 strains to assess the transmissibility of the infection in the two episodes was not performed.

The possible occurrence of reinfections deserves urgent study. A standardized surveillance reporting protocol for cases of suspected reinfections is necessary to better ascertain the burden of this issue, in particular across the EU/EAA countries [22]. Studies on reinfections could be useful to understand the neutralizing response to SARS-CoV-2 infection and the corresponding immunity. However, the absence of a systematic genomic sequencing of positive cases worldwide impedes advances in public health surveillance for the management of the pandemic at the individual and collective level. Further investigation, including a genetic comparison of SARS-CoV-2 strains, would be very useful to better understand the frequency and pathophysiology of SARS-CoV-2 reinfections, particularly in light of the worrisome spreading of SARS-CoV-2 VOC.

## Figures and Tables

**Table 1:** List of mutations of the SARS-CoV-2/human/ITA/APU-POLBA21/2020 strain identified in the first infection (Strain 1) and the SARS-CoV-2/human/ITA/APU-POLBA20/2021 strain identified in the second infection (Strain 2).

	Strain 1	Strain 2
Region	Mutation (s)	Mutation (s)
Spike	D614G	A222V, D614G
N	G204R, R203K	A220V
NS8	-	P30L
NSP3	-	N1116S, T611I
NSP6	-	A54S
NSP12	P323L	P323L
NSP14	-	A323G

## Data Availability

All data regarding the patient and laboratory tests are available from the corresponding author by e-mail request.
